# Diapirs of crystal-rich slurry explain granite emplacement temperature and duration

**DOI:** 10.1038/s41598-023-40805-2

**Published:** 2023-08-23

**Authors:** Alex Copley, Owen Weller, Hero Bain

**Affiliations:** 1 Department of Earth Sciences, University of Cambridge, Cambridge, UK; 2 Present Address: School of Earth Sciences, University of Bristol, Bristol, UK

**Keywords:** Geology, Petrology, Tectonics

## Abstract

The mechanism, temperature, and timescale of granite intrusion remain controversial, with wide-ranging implications for understanding continental growth, differentiation, rheology, and deformation dynamics. In this paper we present a method for determining intrusion emplacement temperature and timescale using the characteristics of the surrounding metamorphic aureole, and apply it to the Skiddaw granite in northern England. The estimated emplacement timescale (0.1–2 Myr) implies magma transport velocities of 1–100 mm/year. At the absent or low melt fractions relevant to our estimated emplacement temperature (580–650 $^{\circ }$C), such velocities are incompatible with pluton formation by successive injections through dykes. Instead, our results indicate the intrusion of a diapir of crystal-rich slurry, solidifying before emplacement, with a rheology governed by the solid crystals. The emplacement depth is likely to be governed by the depth-dependent rheology of the surrounding rocks, occurring close to the brittle-ductile transition. The wider implications of our results relate to (1) the appreciation that much of the chemical and textural characteristics of plutons may relate to pre-emplacement crystallisation at depth, passively transported to higher crustal levels, and (2) an explanation of the difficulty of seismically imaging active plutonism.

The generation, transport and emplacement of granitic magmas play a fundamental role in the chemical differentiation of continental crust, the transport of heat and volatiles, and therefore the rheology, deformation, and geological evolution of the continents^1–3^. However, despite their widespread occurrence throughout the continental crust, the mechanisms governing the formation, transport, and emplacement of granitic melts remain controversial. The relative importance of crustal and mantle melting are poorly known for most intrusive suites^1,4^, and the mechanisms of transport and emplacement are debated^5,6^. In this paper, we focus on the final part of the journey of a far-travelled granitic melt: transport through the mid- to upper-crust, and final emplacement.

As a means to understand the dynamics of granite transport and emplacement, we establish the timescale and temperature of intrusion of a granite pluton. These quantities are difficult to address from observations of intrusions themselves. For example, the ability of magma to transport crystals means that chronometers and thermometers may record partial crystallisation at depth, rather than intrusion, and the nature of the intrusion exposed at the present erosion level may not be representative of the intrusion as a whole. We therefore develop an approach based upon using observations and models of metamorphic contact aureoles to establish the temperature and duration of intrusion. We then use these quantities, in combination with phase equilibria modelling and dynamic models, to establish the possible mechanisms of intrusion.

We demonstrate our method using the Skiddaw granite in northern England (Fig. 1a)^7^. The granite was intruded during the later stages of early-Devonian ‘Acadian’ deformation, which represents the collision of Avalonia and Baltica with Laurentia following closure of the Iapetus ocean basin^8^. The country rocks are the mudrocks of the Ordovician Skiddaw group, which in this area are lithologically homogeneous^9^, and were folded, cleaved, and lightly metamorphosed by the Acadian deformation^10^. The granite outcrops in three locations, which are thought to be on the ‘roof’ of a steep-sided cylindrical pluton. The map pattern of the metamorphic aureole (Fig. 1a), and the geometry of the gravity anomaly produced by the pluton^11,12^, shows that the granite must have a geometrically simple outline and underlie a $\sim$ 6 $\times$ 8 km region at just beneath the current erosion level.

**Figure 1:**
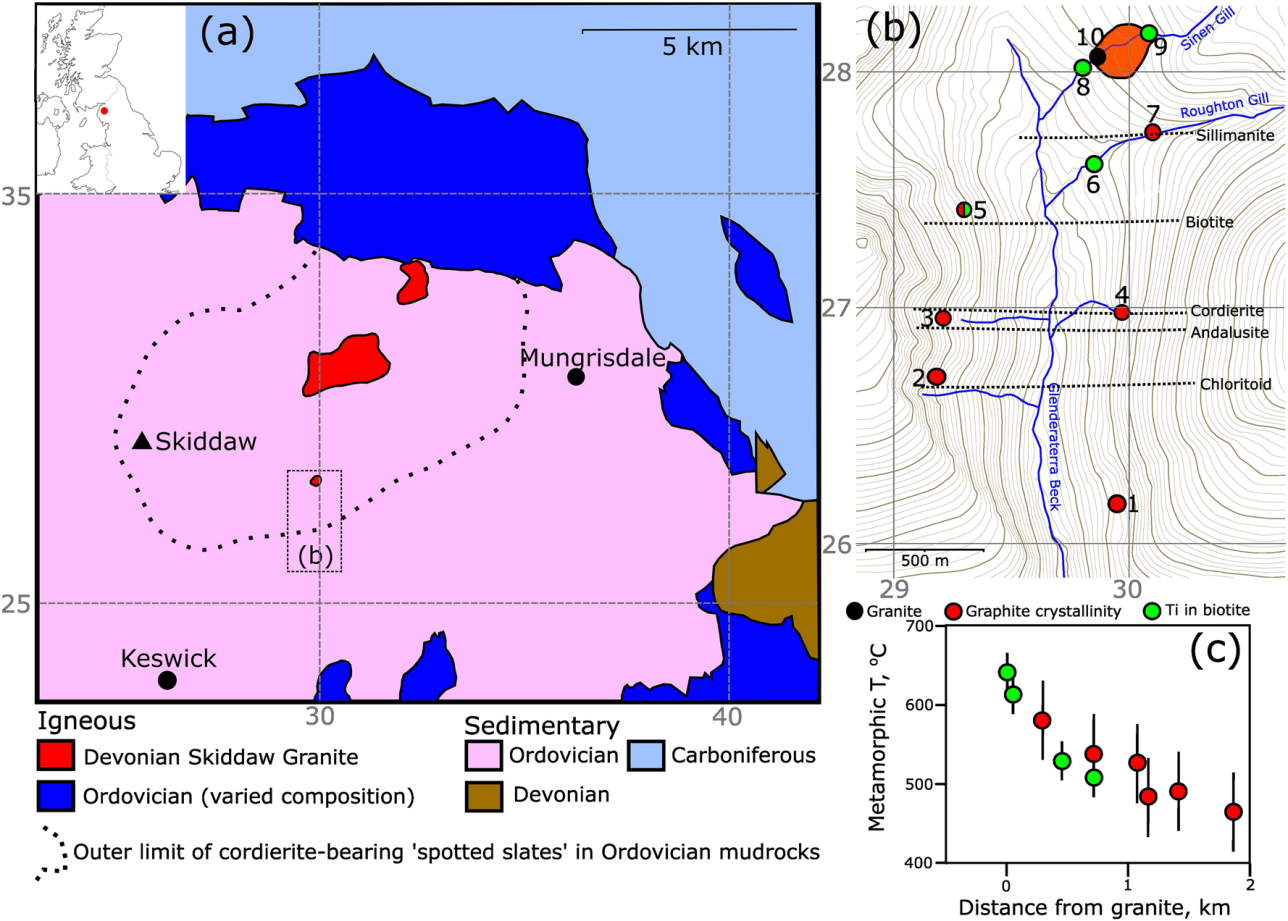
(**a**) Simplified regional geology^11,12^, with the outline of the Skiddaw granite aureole marked. The co-ordinates are the first two figures on the British National Grid. (**b**) Our study area in the Glenderaterra valley, with the samples used for temperature estimates marked, colour-coded according to method. Contour interval is 20 m, and the co-ordinates are the first two figures on the British National Grid. The approximate strike of the isograds is based upon our own observations and previous mapping^7^. Maps were produced using Inkscape (v1.2; https://inkscape.org/). (**c**) Estimated metamorphic temperatures as a function of distance from the granite. Samples 8 and 9 (see **b**) are measured as distance to the granite outcrop (orange shading), and the remainder as distance perpendicular to the metamorphic isograds.

## Results

### Aureole temperature estimates

We sampled the granite, and the well-exposed metamorphic aureole on the southern margin of the intrusion along the Glenderaterra valley. A combination of our observations and previous studies indicate that the aureole spans, in order of increasing proximity to the intrusion, the successive appearance of chloritoid, andalusite, cordierite, biotite, and sillimanite (Fig. 1b)^7,13^. Photomicrographs and descriptions of each sample are provided in the Supplement.

We estimate the maximum temperatures experienced through the metamorphic aureole using two single-mineral thermometers: titanium-in-biotite^14^ and graphite crystallinity^15^. These thermometers are appropriate as the pelites of the Skiddaw group are graphitic, contain rutile and/or ilmenite, and experienced low-pressure metamorphism. Uncertainty associated with these thermometers are ± 12–24 $^{\circ }$C for Ti-in-biotite, and ± 50 $^{\circ }$C for graphite crystallinity^14,15^. See the methods section for details, and the supplement for all analyses.

**Figure 2:**
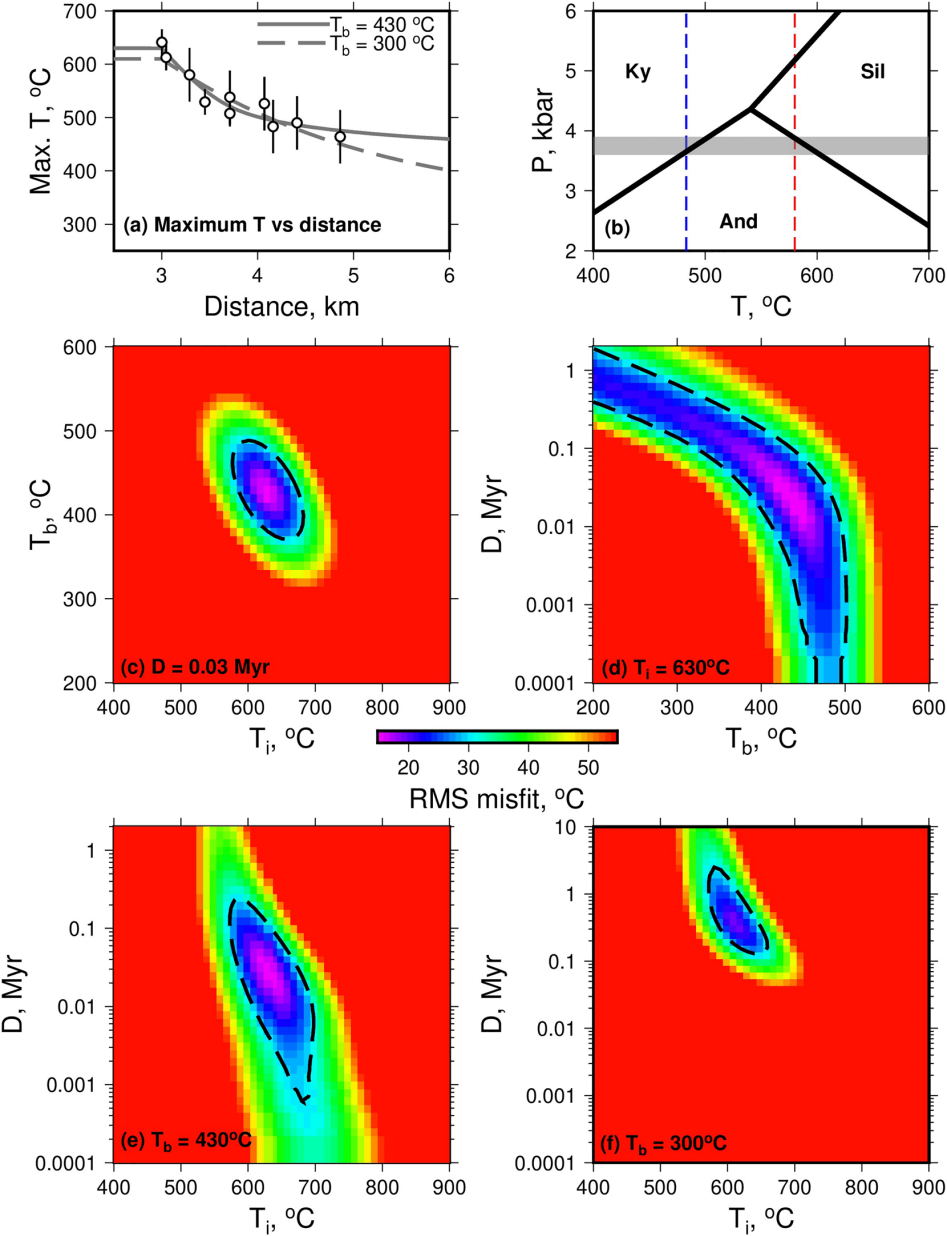
(**a**) Maximum temperature achieved, as a function of position, in the section of the model domain adjacent to the intrusion margin. The points show our temperature estimates, and two of the model solutions are shown as lines (as described in the text). The evolution of temperature through time in these two models is shown in the supplementary information. (**b**) Al$_2$SiO$_5$ phase boundaries, along with our temperature estimates of 580 ± 50 $^{\circ }$C for the sillimanite isograd and 483 ± 50 $^{\circ }$C for the most distal andalusite-bearing sample. The grey shading shows the pressure range compatible with these temperature estimates and mineralogies. (**c–f**) RMS misfit between the model and the data as a function of model parameters, with the parameter held fixed at the best fit value (**c–e**) or the independent estimate (**f**) of that parameter labelled in the bottom left corner of each plot.

Figure 1c shows the results of the contact metamorphic temperature estimates as a function of distance from the outcrop of the granite, showing the expected decrease with distance, and agreement between the two techniques in the regions where both could be used. The sillimanite isograd was defined in the field at the outcrop of sample 7, which recorded a metamorphic temperature of 580 ± 50 $^{\circ }$C. Andalusite was present more distant from the intrusion (Figure 1b), and sample 3 (close to the andalusite isograd) recorded a temperature of 483 ± 50 $^{\circ }$C. The presence of sillimanite and andalusite at these temperatures indicates pressures during metamorphism of $\sim$ 3.75 kbar (grey shaded band on Fig. 2b), equivalent to a depth of $\sim$14 km for a crustal density of 2800 kg/m$^3$. This estimate is consistent with the 3–4 kbar previously suggested for aureoles that display the mineral sequence we have observed^16^.

### Granite intrusion characteristics

We employ a model of thermal diffusion^17,18^, taking into account the temperature-dependence of the thermal parameters^19^, to estimate the granite intrusion temperature and duration using our observations from the metamorphic aureole. The model is described in the “Methods” section, and the free parameters are the background temperature of the country rocks ($T_b$), the temperature at which the granite was intruded ($T_i$), and the duration of intrusion over which continued flux of granite through the crustal level now exposed at the surface resulted in a continued supply of heat (*D*). We approximate this continued heat supply as a constant temperature within the granite during this time period at this crustal level. This temperature will capture the effects of both heat transported by the granite, and any latent heat released during crystallisation (although we expect this latter effect to be minor, as we suggest below that the granite was mostly or entirely solid during emplacement). During intrusion, heat transfer between the granite and the country rocks acts to equalise the temperatures of the granitic body and the country rocks adjacent to the intrusion. The commonly-used approximation of the temperature at the contact being the mean of the intrusion and background country rock temperatures only applies for the case of instantaneous intrusion, which is unrealistic for a large pluton. In the supplement, we show forward models which indicate that during intrusion at geologically viable rates^1,6^, the combination of the temperatures we have estimated in the aureole with our modelling approach will accurately constrain the temperature of intrusion.

In Fig. 2, our temperature estimates are used to assess the misfit between the models and the data as a function of $T_b$, $T_i$, and *D*. The dashed lines show an RMS misfit of 30 $^{\circ }$C (intermediate between the accuracies of the two methods of estimating metamorphic temperatures), and all areas that are red show models that fit the data worse than a constant temperature with the same value as the mean of the data (i.e. the presence of the intrusion degrades the fit to the data). The best-fitting models have misfit values of 19 $^{\circ }$C. There are well-defined misfit minima in (c) and (e), showing limited tradeoffs between these pairs of model parameters. However, (d) shows a tradeoff between $T_b$ and *D*. For the best-fitting model, shown by the solid grey line in (a), $T_i$ = 630 $^{\circ }$C, $T_b$ = 430 $^{\circ }$C, and *D* = 30 Kyr. In regions unaffected by the intrusion of the Skiddaw granite, studies of illite crystallinity have suggested that the region experienced upper anchizone to lower epizone conditions during Acadian deformation^10^, during which the pluton was emplaced^8^. It is not straightforward to relate this observed low metamorphic grade to a temperature, but $\sim$ 300 $^{\circ }$C is a likely value. As shown on (f), if $T_b$ is set to 300 $^{\circ }$C, the best-fitting $T_i$ is 610 $^{\circ }$C and *D* is 400 kyr, and this model is shown by the dashed grey line in (a). If the edge of the intrusion is inclined, rather than vertical, then the distance between each sample and the intrusion margin will be lower than those used above. To investigate this effect, we have re-run the inversions with the distances re-calculated using a dip of 60$^{\circ }$ for the intrusion margin. These results are shown in the supplement, and indicate little difference to those in Fig. 2. Considering all models that can match our observations, and with $T_b$ = 300 $^{\circ }$C, we can constrain $T_i$ to be 580–650 $^{\circ }$C, and *D* to be 0.1–2 Myr.

Our estimated intrusion timescale can be used to place limits on the possible granite intrusion velocities. As a minimum bound, our longest estimated duration (2 Myr) could represent the time required for the granite to travel by a distance larger than the width of the mineralogically-distinct thermal aureole ($\sim$ 2 km), so that the granite moved from a location that does not thermally affect the exposed rocks to one that does. In this case, the velocity would be 1 mm/year. At the other extreme, our shortest estimated duration (0.1 Myr) could represent the time taken to transport granite a greater distance, from the $\sim$10 km deep ‘Lake District Batholith’ thought to underlie the region^8,20^. In this case, the velocity would be 100 mm/year. Models with a higher value for $T_b$ correspond to shorter intrusion durations (Fig. 2e), resulting in faster velocities, which would not affect our conclusions outlined below regarding the emplacement mechanism. We return below to the question of whether these could be lower bounds, because of the pluton being formed by multiple sub-injections of fast-moving granite spaced out in time.

### Melt fraction during emplacement

**Figure 3:**
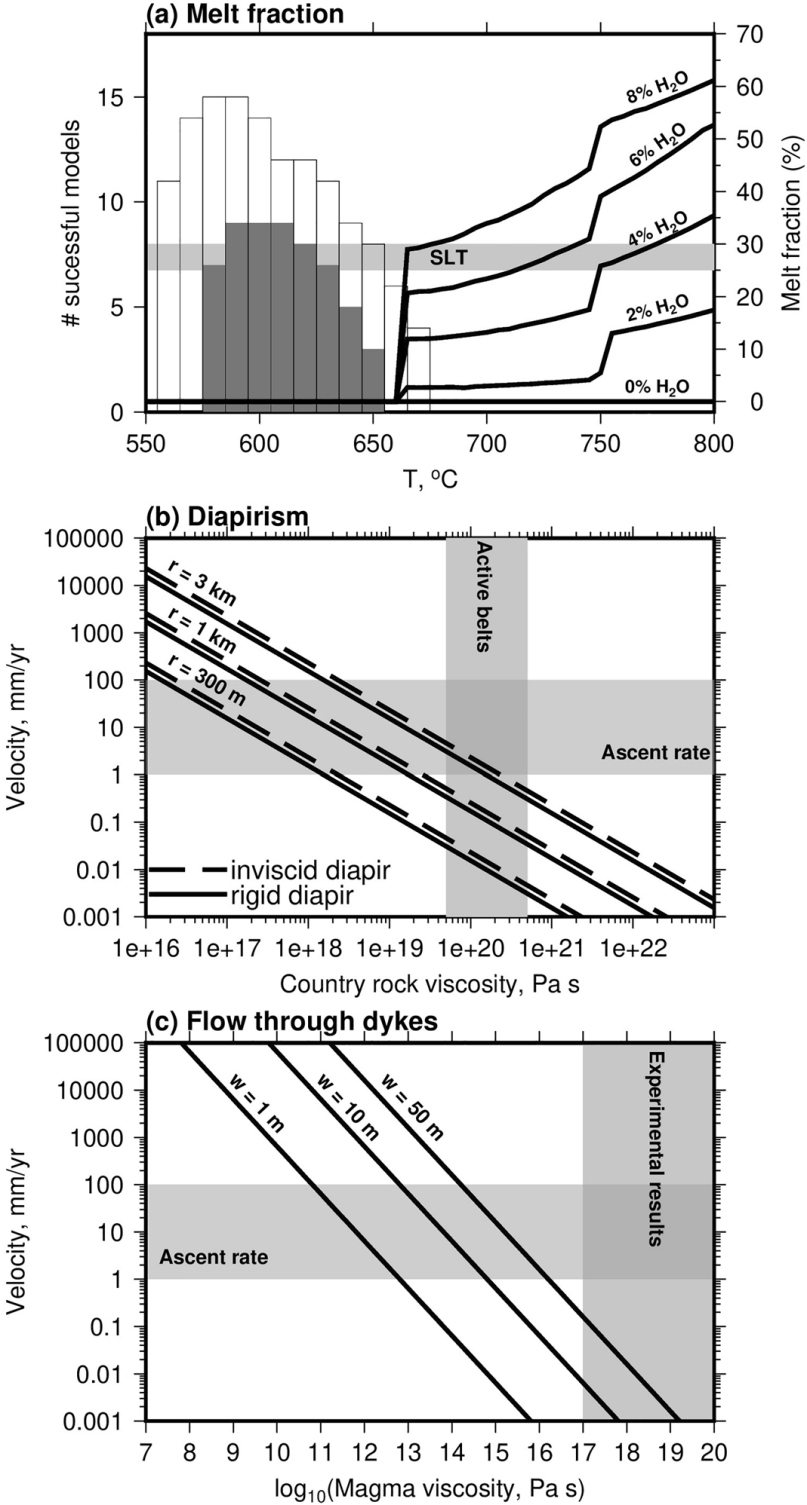
(**a**) Melt fraction versus temperature for the bulk composition of the Skiddaw granite at a pressure of 3.75 kbar and at a range of water contents (labelled in mol%). Histograms show the number of models from Fig. 2f that can fit the data to within an RMS misfit of 30 $^{\circ }$C (solid bars) or 38 $^{\circ }$C (thin lines), for $T_b$ = 300 $^{\circ }$C and the model space sampled at increments of 10 $^{\circ }$C in $T_i$ and 0.1 in log$_{10}$(*D*). (**b**) Rigid and inviscid diapir ascent rates as a function of radius and country rock viscosity. The shading shows the effective viscosity of active fold-thrust belts of similar lithology to the Skiddaw Group^36,37^. (**c**) Ascent rate in dykes of various widths, as a function of the effective viscosity of the liquid-solid magma mixture. The shading shows viscosities within three orders of magnitude of experimentally-derived solid mineral flow laws at relevant conditions^38^.

Figure 3a shows the results of phase equilibria modelling to estimate the melt content of the intrusion as a function of temperature, at our estimated pressure of intrusion, using the bulk composition of a collected granite sample and considering a range of water contents. These estimates were calculated using MAGEMin^21^, which uses the most up-to-date thermodynamic database^22^ and activity-composition models of relevance to granitic bulk compositions^23,24^. The melt contents as a function of temperature for the range of bulk compositions are similar, and feature two steps: a higher-temperature step associated with changing biotite mode, and a lower-temperature step at the wet solidus. The only notable difference between the bulk compositions is in the extent of melting at the latter, which is controlled by the water content. Also shown are the estimated intrusion temperatures, for a background temperature of 300 $^{\circ }$C, and which fit the data to within an RMS misfit of 30 $^{\circ }$C (solid grey, as shown as contours on Fig. 2) and 38 $^{\circ }$C (thin lines, chosen to be double the misfit of the best-fitting model, as an extremely conservative bound). For all except 8% of the models for this conservatively-high misfit bound, the estimated intrusion temperature is lower than the solidus temperature, implying that the granite was solid when emplaced.

For the few models that overlap the solidus temperature, for water contents of less than 8 mol%, the melt fraction would have been below the threshold for melt-dominated deformation (sometimes called the ‘solid-to-liquid transition’) of 25–30%. Below this threshold, the effective viscosity of a mixture of melt and crystals is within $\sim$3 orders of magnitude of the viscosity of the crystals deforming by solid-state creep^25–27^. The mineralogically-bound water in the granite sample is 1.51 mol% (see supplementary information), providing a lower limit on the relevant water contents to consider. However, the water content of the melt could have been higher, and water may have been driven off as a separate phase during crystallisation. The lack of pervasive veining in the country rocks near the granite contact implies that such a process did not occur in our study area, or occurred in an earlier stage of the transport of the granite, before final emplacement. More fluid-related alteration is observed along the north-eastern edge of the granite body^7,9^. However, the volume of fluid-related alteration in that area is small compared to the volume of the granite itself, there is no significant change in the major element chemistry of the country rocks near the granite^9^, and the location of the present erosion level, near the roof of the granite, will skew our observations in favour of higher fluid contents. When combined, these observations imply limited fluid presence in, or release by, the granite, as also observed in some other ‘late orogenic’ granites^28^. We therefore infer that during pre-emplacement transport the granite is likely to have been a crystal-rich slurry (Fig. 3a), with a rheology governed by the solid crystals. Although some studies have suggested that peralkaline [mol (Na + K) > Al] melts may persist to temperatures as low as $\sim$ 500 $^{\circ }$C^29^, such a situation is unlikely to be relevant to Skiddaw due to the peraluminous composition ([mol Al > (Na + K + 1/2Ca)]; see Supplementary Data Tables). If a small proportion of late-stage peralkaline melt were added to the intrusion, it would need to be minor to not affect the overall intrusion composition, which would leave our conclusions regarding the nearly, or entirely, solid nature of the pluton during emplacement unchanged.

## Discussion

### Emplacement mechanism

The results described above show that for transport through the mid- to upper-crust and during emplacement to form the pluton, the granite was a crystal-rich slurry, becoming entirely solid before final emplacement. The pervasive undulose extinction and incipient sub-grain formation in quartz grains in the granite attest to significant solid-state deformation, as would be expected in this scenario (see Supplemental Material). The major-element zoning in the plagioclase crystals, featuring core-to-rim increases in albite content (see Supplemental Material), may imply a protracted period of cooling at depth, before emplacement as a dominantly solid body. We can use our results regarding the temperature and duration of intrusion to investigate the dynamics of the granite transport and emplacement.

**Figure 4:**
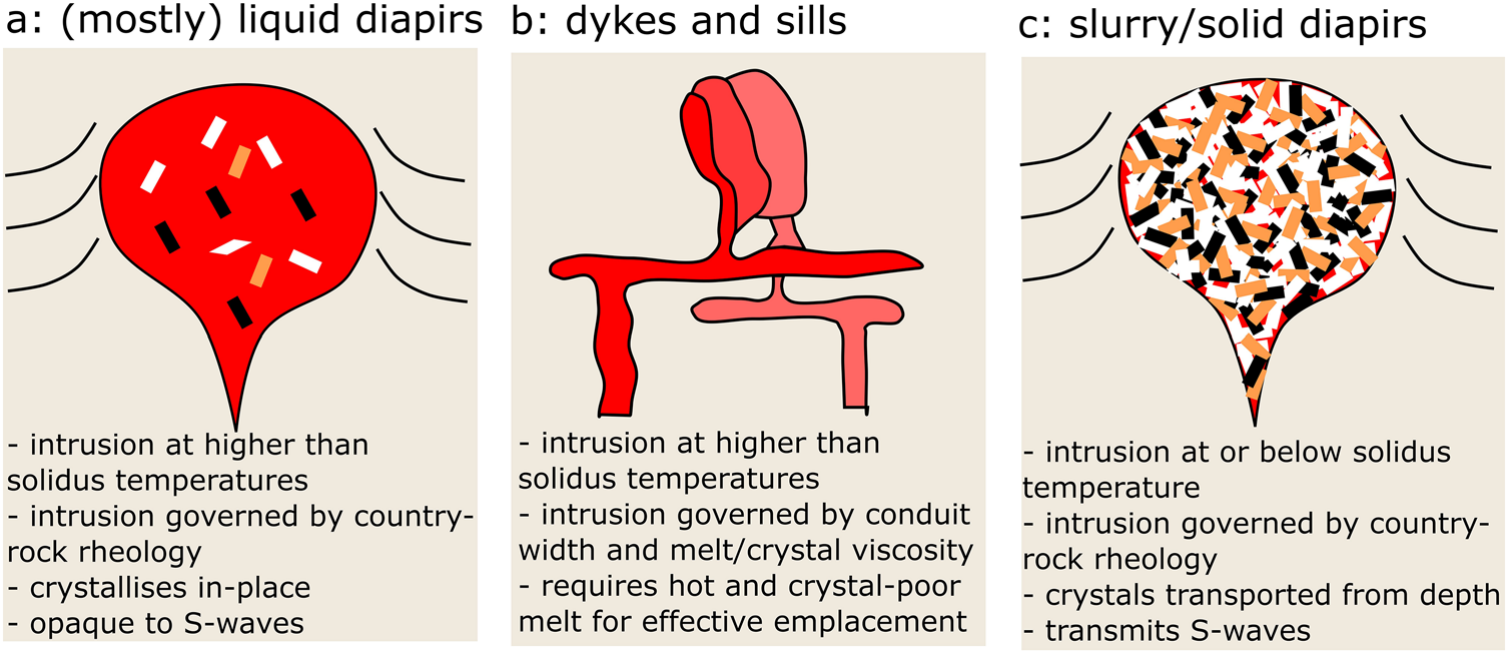
Possible mechanisms and characteristics of granite emplacement.

One possible mechanism for granite emplacement is as a diapir^30–32^ (Fig. 4a,c). The rate of ascent of a diapir is mainly controlled by its size, the density contrast with the surrounding rocks, and the effective viscosity of the surrounding rocks. The rheology of the diapir itself is less important, and the rate of ascent of an inviscid diapir only differs by a factor of 3/2 from that of an entirely rigid one, due to the deformation of the country rocks surrounding the diapir dominating the dynamics^33,34^. Figure 3b shows that for a diapir the size of the Skiddaw granite (radius of 3 km), and a density contrast of 250 kg/m$^3$ with the surrounding rocks (based upon previous density measurements and gravity modelling^35^), the necessary ascent rates can be achieved for a country-rock viscosity of 10$^{18}$–10$^{20}$ Pa s (see “Methods” section for details of the calculation). Part of this range agrees with the viscosities estimated for present-day deformation belts dominated by similar sedimentary lithologies^36,37^, showing that the emplacement of a diapir in the available timescale is viable, regardless of whether the granite is molten or solid. Because of the dependence of diapir ascent rate on the square of the radius, it would not be possible to form the pluton in the required timescale by multiple, smaller, sub-intrusions (Fig. 3b).

A popular alternative view to diapirism is the formation of plutons by protracted melt flow through a (possibly sheeted) network of dykes and sills (Fig. 4b)^1^. In this case, the ascent rate depends upon the density contrast with the surroundings, the viscosity of the flowing crystal-liquid mixture, and the width of the dyke (see “Methods” section). Figure 3c shows that our inferred ascent rate is not viable for geologically plausible dyke widths, the same density contrast as used above, and viscosities of the liquid-crystal mix that are within 3 orders of magnitude of the relevant ‘solid’ mineral flow laws, as discussed above (e.g. > 10$^{20}$ Pa s for ‘wet’ feldspar deforming by dislocation creep at a temperature of 650 $^{\circ }$C and a strain rate of 10$^{-13}$ /s^38^).

We now consider whether it would be possible to form the Skiddaw pluton in a composite manner, by the injection of multiple batches of granite^17^, and whether they would all be required to be at sub-solidus temperatures. The metamorphic temperatures recorded by the samples immediately adjacent to the pluton require that the outer part of the granite was intruded at our estimated sub-solidus temperature, and the gradient of decay of metamorphic temperature with distance through the aureole places constraints upon the timescale of that sub-solidus intrusion. Could a later intrusion of granite in the centre of the pluton have occurred, and could it have been at supra-solidus conditions? From a thermal perspective, Fig. 2 shows that the aureole temperature perturbation decays to around half of the maximum within 0.5–1 km of the intrusion margin. Therefore, a hot second batch of granite intruded within the first sub-solidus intrusion would be invisible in the thermal aureole provided that it was more distant than $\sim$ 1 km from the intrusion margin, and provided that the outer intrusion had cooled sufficiently before the time of the second intrusion so that it’s outer edge did not exceed the initial intrusion temperature. The timescale for sufficient cooling is given by $\tau = l^2 / \pi ^2 \kappa$, where $\tau$ is the characteristic timescale for diffusive cooling, *l* is the width of the initial intrusion, and $\kappa$ is the thermal diffusivity. In this situation, the maximum temperature experienced in the aureole after the second intrusion would be everywhere lower than after the initial intrusion, and only within the granite itself (where we cannot estimate metamorphic temperatures) would the intrusion of an inner, hotter, granite induce higher temperatures than were experienced during the first intrusion. However, further constraints are provided by the mechanics of intrusion. In the case of a thermally ‘invisible’ later intrusion in the centre of the pluton, our estimated temperatures and timescales will only be sensitive to the timescale of the initial intrusion, which is what sets the distribution of metamorphic temperatures in the country rocks in this scenario. For the initial sub-solidus intrusion, we have shown above that flow through a network of dykes is inconsistent with the observed timescale of intrusion. For diapiric intrusion, because the rate of ascent depends upon the square of the radius (see “Methods” section), that mechanism is only possible for intrusions that are approaching the size of the entire Skiddaw granite. For example, an intrusion radius of 1 km can be seen from Fig. 3b to be inconsistent with the country rock viscosities that characterise modern-day mountain belts of similar lithologies to the Skiddaw region. An intrusion radius of 2 km would be just consistent with the very lowest combination of country rock viscosity and granite ascent rate. Therefore, we can conclude that any thermally-invisible later intrusion of granite in the centre of the pluton would be required to form only a small proportion of the volume of the pluton as a whole, otherwise the initial sub-solidus emplacement would not have been mechanically feasible. For example, a 1 km radius cylindrical core would have 12.5% of the volume of a surrounding 2 km wide annulus of the same vertical extent.

### Controls on the depth of emplacement

Our results indicate that the Skiddaw granite was emplaced as a solid diapir, after transport as a crystal-rich slurry (Fig. 4c). Such an emplacement mechanism explains the near-spherical outcrop of the aureole, and so intrusion (Fig. 1), because diapirs form circular shapes in plan view^39^. The emplacement depth was not limited by the neutral buoyancy level, as the negative gravity anomaly over the granite^11,12,35^ indicates it is less dense than the surrounding rocks. The intrusion level was also not limited by the solidus temperature, because the rate of rise of a solid diapir is close to that of an inviscid one, and the emplacement temperatures we have estimated imply that the pluton continued to rise after it had solidified^34^. Instead, the emplacement level is likely to have been governed by the rheology of the country rocks. Our estimated intrusion depth of $\sim$ 14 km lies close to the brittle-ductile transition in present-day mountain belts characterised by similar geometries and lithologies^36,37,40^. Diapir ascent therefore likely ceased when the surrounding country rocks were unable to deform in a ductile manner to accommodate the ascent of the diapir.

Our estimated depth of emplacement of $\sim$ 14 km is shallower than is seen in many models^41,42^, and we suggest that this feature is related to the country rock lithology and rheology, and their control on the depth of the brittle-ductile transition. The observationally-constrained effective viscosities used above^36,37^ are from present-day mountain belts that are dominantly composed of thick sedimentary sequences, and contain significant quantities of mudrocks, as is the case for the country rocks at Skiddaw (which are almost entirely mudrocks). The estimated viscosities are lower than predicted by the experimentally-derived flow laws that are often used in numerical models, which generally focus on more experimentally-amenable, and stronger, lithologies (e.g. pure quartzite, or igneous and high-grade metamorphic assemblages^43^). Given the dominant role that country rock rheology, and the resulting depth to the brittle-ductile transition, play on diapir ascent, our findings and the previous work together imply that emplacement depth will be at least partially governed by the lithological controls on depth-dependent rheology.

It is likely that the space required for the intrusion of the Skiddaw diapir was produced by the deformation of the surrounding country rocks. Unfortunately the level of exposure at Skiddaw means it is not possible to directly test this assumption: our observations are along a valley with linear outcrop bands, but the surrounding grassy fellsides prevent the two-dimensional map pattern of the structures being accurately determined. Given the complex polyphase deformation history of the region^11,12^, it is therefore not possible to disentangle the effects of granite intrusion from the prior and subsequent deformation fabrics. The aureole of the nearby Shap granite shows foliations that wrap around the margins of the intrusion^8^, consistent with diapiric emplacement, but that intrusion lacks the exposure required to obtain a profile of temperature versus distance as we achieved at Skiddaw.

### Wider implications

It remains to be seen how representative the Skiddaw granite is of plutons in general. However, our method provides a means to survey the intrusion parameters of a wide range of plutons, and makes a testable prediction of emplacement depth as a function of country rock lithology: stronger surroundings, with a deeper brittle–ductile transition, should result in deeper pluton emplacement. Whilst some mid- to lower-crustal granite exposures clearly represent flow through distributed dyke networks^44^, and Skiddaw may be underlain by such a network in the lower crust, further work is required to explore the link between these plumbing systems and the mechanism and characteristics of final emplacement.

Dating results have been used to suggest the formation of plutons by the sequential emplacement of multiple smaller bodies, resulting in a wide spectrum of ages^45–48^. However, our results presented above suggest an alternative explanation for these observations. If a granite is intruded as a solid body or a very crystal-rich slurry, then high-temperature thermochonometers may be recording the prior crystallisation at depth, rather than the emplacement of the granite. In this case, pre-existing age contrasts inherited from pre-intrusion crystallisation will be passively transported (with limited stirring due to the high viscosity) to higher crustal levels, and will not be recording the timing of emplacement of the granite^8^. The geochemically or chronologically diverse portions of large plutons may therefore represent the signature of lower- to mid-crustal crystallisation patterns that have been transported to the upper crust in coherent diapirs, rather than being the result of a composite method of emplacement. Likewise, techniques that estimate the crystallisation temperature of minerals may record this pre-emplacement crystallisation, rather than the subsequent solid-state intrusion. A further implication of our results is an explanation for the difficulty of seismically imaging active granitic diapirs—the almost entirely solid slurry means that shear waves can propagate through them.

## Methods

### Analytical details

All analytical techniques were conducted in the Department of Earth Sciences at the University of Cambridge. For sample 10, a full thin section phase map, with associated mineral abundances, was calculated by ‘quantitative evaluation of minerals by scanning electron microscopy’ (QEMSCAN), using a Quanta650F scanning electron microscope, with a 10 $\upmu$m pixel size.

Mineral compositions of biotite in samples 5–9, and all major phases in sample 10, were measured by Electron Microprobe Analysis (EMPA) using a Cameca SX100. Analyses were carried out with a 20 kV acceleration voltage, a 2–3 $\upmu$m beam diameter, and a 20 nA probe current. Mineral cation totals were calculated using AX^49^, which calculates mineral compositions based on standard number of oxygens per formula unit (pfu) and estimates Fe^3+^ based on stoichiometric criteria. Any analyses indicating partial retrograde breakdown of the biotite were excluded. All remaining analyses are reported in Table S1. Average cation totals for all phases in the granite sample 10 are reported in Table S2.

Raman spectra of carbonaceous material were collected for samples 1–9 using a LabRam300 Horiba Raman microspectrometer with a 532.05 nm wavelength laser. The laser was focussed on the sample by a $\times$ 50 magnification objective (numerical aperture = 0.50) and the spot size at the sample surface was $\sim$ 2 $\upmu$m in diameter. The laser power was set at the source at 250 mW. The laser wavelength was eliminated by a notch filter and the signal was dispersed using a 600 grooves mm$^{-1}$ grating and analysed by a CCD detector. The spectrometer was calibrated before each session using the 528 cm$^{-1}$ peak of a silicon standard. Between 6 and 15 spectra were acquired for each sample and averaged to determine a representative R2 ratio. Average spectra are shown in the Supplement for all samples that yielded consistent measurements.

### Thermometry

Of the nine metasedimentary samples collected from the aureole, five samples (samples 5–9) were collected inwards of the biotite isograd (Fig. 1) and thus amenable to Ti-in-Bt thermometry. Of these five samples, sample 7 contained altered biotite and was excluded from analysis. Biotite analyses are reported in Table S1. Ti-in-biotite temperatures were calculated using the mean of each sample set, yielding temperatures of 507 $^{\circ }$C for sample 5 (*n* = 13), 529 $^{\circ }$C for sample 6 (*n* = 10), 613 $^{\circ }$C for sample 8 (*n* = 11), and 641 $^{\circ }$C for sample 9 (*n* = 18). The Mg# (mol Mg/(Mg+Fe)) of the results range from 0.237 to 0.301 which spans the lower bound of 0.275 for which the Ti-in-Bt thermometer is calibrated^14^. However, there is a single consistent relationship between Ti content, Mg#, and temperature for Mg#’s ranging from 0.275 to 1.0, and our results for biotites within the calibrated range agree with those from the lowest Mg# we have measured (0.237).

All samples contained carbonaceous material, but only samples 1–5 and 7 yielded reproducible spectra. The results show a systematic decrease in the relative area of the defect band (R2 ratio) on approach to the pluton, consistent with progressive graphitization and increasing temperatures through the aureole. As the spectra were acquired at the polished sample surface, the R2 ratio was corrected using the measurements of Ref.^50^, which defined a linear relationship between polished and unpolished graphite. This correction increased the graphite temperatures by 24 to 60 $^{\circ }$C, as shown in Table S3.

Although phase equilibria modelling is used below to estimate plausible melt fractions in the granite, we do not apply the technique to model the aureole assemblages, owing to the poor calibration of activity-composition (*a*-*X*) models for pelites at low pressures and sub-solidus temperatures^51–53^.

### Phase equilibria modelling

A bulk composition for sample 10 was calculated by combining the QEMSCAN-derived vol% estimates with EMPA-derived compositional analyses of the major phases (see supplementary data spreadsheet). Alteration phases (e.g. sericite) were subsumed into their host phases (e.g. plagioclase) to determine the vol% prior to retrogression, to mitigate possible effects related to metasomatism. As plagioclase exhibited zoning, line profiles were taken through selected grains and a radially-weighted average composition determined assuming a cubic morphology. For all other phases that exhibited no zoning, a representative compositional analysis was used. The H content of biotite was set assuming Ti-protonation (i.e. H = 2 - 2Ti) to be consistent with the biotite model considered in ref.^23^. The determined bulk composition (wt%) is: SiO$_2$ = 68.49, TiO$_2$ = 0.48, Al$_2$O$_3$ = 16.21, Fe$_2$O$_3$ = 0.01, FeO = 2.64, MnO = 0.07, MgO = 1.07, CaO = 1.95, Na$_2$O = 4.33, K$_2$O = 4.31, H$_2$O = 0.42. For phase equilibria modelling, the composition was converted to a MAGEMin compatible input, which lists mol Fe$_2$O$_3$ as ‘O’, and FeO$^t$ = FeO +2O (as per thermocalc). The water content of the sample was varied, and minor Mn was ignored as this component is not in the model system of ref.^23^. The Supplementary Data spreadsheet lists all of the compositions used as input for the modelling.

### Numerical model

We employ a model of thermal diffusion^17,18^ to estimate the intrusion temperature and duration, using our observations from the metamorphic aureole. Based upon the map pattern of the aureole, and gravity inversions suggesting a steep-sided cylindrical shape to the intrusion^20,35^, we produce our model in a cylindrical coordinate system, with temperature only varying in the radial direction:

1 $$\begin{aligned} \rho \frac{\partial {C(T) T}}{\partial {t}} = \frac{1}{r} \frac{\partial }{\partial {r}}\left( r k(T) \frac{\partial T}{\partial r} \right) , \end{aligned}$$

where *T* is temperature, *t* is time, *r* is the radial co-ordinate, and $\rho$ is density. We used existing estimates of the temperature-dependence of the heat capacity (*C*) in crustal rocks, and along with the temperature dependence of thermal diffusivity also calculated the temperature-dependent conductivity (*k*)^19^. In the model, the region is initially at a constant background temperature ($T_b$), and an intrusion with the observed radius of 3 km (Fig. 1) appears with an intrusion temperature ($T_i$), which is maintained at that value for an intrusion duration (*D*; to simulate the continued input of hot granite) before being able to cool. The country rock begins to heat up from the moment the intrusion appears. Unlike the northern edge of the intrusion, where there are regions of silicification and mineralisation^7,9,13^, there is minimal field or textural evidence for fluid flow through the region of our sample traverse, with limited veining and only sporadic retrogression. We therefore do not include the effects of heat advection by fluids in our models.

Using the product rule, we re-write the above equation as

2 $$\begin{aligned} \rho \frac{\partial {C(T) T}}{\partial {t}} = k(T) \frac{\partial ^2 T}{\partial {r^2}} + \frac{k(T)}{r} \frac{\partial T}{\partial r}. \end{aligned}$$

We solve this equation using finite differences, employing operator splitting^54^. Both terms are solved using a Crank-Nicholson (joint implicit-explicit) scheme^54^. The temperature-dependence of the thermal parameters are taken into account by iterating over each time-step until a consistent set of temperatures and thermal parameters at the start and end of the timestep are achieved^55^.

The boundary conditions are zero temperature gradients (i.e. no heat flux) at each end of the model domain. For the *r* = 0 boundary this condition represents the cylindrical symmetry of the model setup. The far end of the model is placed distant enough from the intrusion that the nature and location of the boundary condition do not affect our results.

### Emplacement rates

The ascent velocity of a diapir is given by

3 $$\begin{aligned} u = \frac{1}{3} \frac{\Delta \rho g r^2}{\eta _c} \left( \frac{\eta _c + \eta _d}{\eta _c + \frac{3}{2} \eta _d} \right) , \end{aligned}$$

where *u* is the terminal velocity of a spherical body, $\Delta \rho$ is the density contrast between the diapir and the country rock, *g* is the acceleration due to gravity, *r* is the diapir radius, $\eta _c$ is the country rock viscosity, and $\eta _d$ is the diapir viscosity^6,33^.

The rate of flow through a dyke, driven by the density contrast with the surroundings, is given by

4 $$\begin{aligned} u = \frac{\Delta \rho g d^2}{12 \eta _m}, \end{aligned}$$

where *u* is the average flow rate in the dyke, $\Delta \rho$ is the density contrast between the magma-crystal mixture and the country rock, *d* is the dyke width, and $\eta _m$ is the effective viscosity of the magma-crystal mixture^6^.

### Supplementary Information

Supplementary Information.

Supplementary Tables.

## Data Availability

All compositional analyses are provided in the supplemental information. Specimens are available from the authors on request.
