# Supplementary material for: Diapirs of crystal-rich slurry explain granite emplacement temperature and duration
Source: Sci Rep. 2023 Aug 23;13:13730. doi: 10.1038/s41598-023-40805-2 (PMC10447574; doi:10.1038/s41598-023-40805-2)
Supplement: Supplementary file 1 — Supplementary Information. [file 41598_2023_40805_MOESM1_ESM.pdf]

Supplementary material for 'Diapirs of crystal-rich slurry explain granite emplacement temperature and duration'

Alex Copley, Owen Weller, and Hero Bain

August 9, 2023

1 Sample descriptions

Nine samples were collected from the aureole, labelled 1–9 in order of proximity to the granite, and one sample was collected from the granite body, labelled 10. Supplementary Figures 1 and 2 show petrographic images of all samples. A brief description of each is given below.

Sample 1 dominantly comprises quartz, muscovite and chlorite, with accessory apatite, monazite, rutile, tourmaline and opaques. The sample is very fine grained, and exhibits compositional banding between quartz-rich and quartz-poor domains. Aligned mica laths define a foliation that is sub-parallel to the compositional banding. Both the sedimentary layering and primary foliation are folded, yielding a crenulation cleavage axial planar to the microfolds, which is best developed in the mica-rich layers. Larger chlorite-muscovite books are variably oriented in the microlithon domains.

Sample 2 dominantly comprises quartz, muscovite and chlorite, with accessory chloritoid, apatite, monazite, rutile, and opaques. The sample is very fine grained, and exhibits compositional banding between quartz-rich and quartz-poor domains. Aligned mica grains define a penetrative foliation that is discordant with the compositional banding. Chloritoid is present as small grains ($\sim$50 μm) that are randomly orientated throughout the matrix.

Sample 3 is graphite-rich and comprises randomly orientated, mm-sized chiastolite-type andalusite porphroblasts in a very fine grained matrix dominantly comprising quartz, muscovite and chlorite with accessory apatite, rutile and opaques. The sample exhibits a foliation sub-parallel to compositional banding, with an incipient crenulation cleavage developing axial-planar to open micro-folds. Andalusite grains are commonly altered to an aggregate of white mica and quartz, with chlorite along the margins. The grains exhibit classic chiastolite microstructures, with basal sections trapping graphite, whereas prism faces exclude graphite.

Sample 4 features cordierite and andalusite porphyroblasts that overgrow a very fine grained matrix dominantly comprising quartz, muscovite and chlorite with accessory rutile, tourmaline and opaques. The sample exhibits a penetrative foliation that is sub-parallel to

weak compositional banding. Andalusite is commonly altered, and features graphite-rich margins. Cordierite is also commonly pinitised.

Sample 5–7 all feature abundant cordierite and biotite porphyroblasts, with lesser andalusite porphyroblasts, which overgrow a fine-grained matrix dominantly comprising quartz, muscovite and additional biotite, with accessory apatite, plagioclase, rutile, zircon and opaques. Andalusite grains commonly feature graphite-rich accumulations adjacent to prismatic faces, and all samples exhibit a penetrative foliation that is sub-parallel to weak compositional banding. Samples 5 and 6 are relatively unaltered, whereas sample 7 is pervasively altered, with andalusite pseudomorphed by aggregates of white mica and quartz, cordierite grains pinitised, and biotite consistently yielding low EMPA totals and low K content indicative of retrogression. The sillimanite isograd was defined in the field at the location of sample 7, but the associated thin section did not capture any of the sillimanite needles visible in outcrop, possibly due to the alteration.

Sample 8 comprises cordierite and andalusite porphyroblasts that overgrow a matrix rich in biotite, muscovite and quartz, with accessory tourmaline, apatite, plagioclase and opaques. Andalusite grains are notably anhedral, and are assumed both resorbed and metastable with respect to fine sillimanite needles nucleating in biotite. Aligned mica define a penetrative foliation sub-parallel to thin-section scale compositional banding.

Sample 9 is the coarsest and most quartz-rich sample, and features abundant cordierite porphyoblasts that overgrow a matrix rich in biotite, muscovite, and quartz, with accessory apatite, rutile, plagioclase and opaques. Two aluminosilicate phases are also present: sillimanite, as sprays of needles that radiate from biotite grains; and andalusite, which features highly corroded and irregular grain boundaries and is presumed metastable. Cordierite is highly poikiloblastic and exhibits sector trilling. Quartz forms polygonal grains and micas are relatively poorly aligned, both indicative of a hornfelsic texture.

Sample 10 is a coarse-grained, porphyritic granitoid comprising partially sericitised plagioclase with secondary epidote (45.9 vol.%), quartz (23.4 vol.%), K-feldspar (18.6 vol.%), and biotite with secondary chlorite and titanite (11.9 vol.%). Apatite, zircon and opaques are present as accessory phases. Plagioclase grains contain oscillatory-zoned cores that vary from $\sim$0.50–0.70 albite content (Na/[Na + Ca + K]), mantles that feature an increase in albite content up to 0.86, and thin albitic rims (An $\sim$0.95). Quartz grains exhibit undulose extinction, irregular grain boundaries, and incipient sub-grain formation. K-feldspar grains feature perthite textures. Biotite laths exhibit pleochroic haloes from abundant zircon inclusions, and feature secondary chlorite and titanite along basal cleavages. On a quartz-alkali feldspar-plagioclase diagram, the thin section abundances plot in the granodiorite field, near to the border with a monzogranite.

2 Sample composition information

All mineral and whole-rock composition data is provided in the associated spreadsheet of data.

3 Raman Spectra

Average spectra are shown in Figure 3 for all samples that yielded consistent measurements.

4 Forward model of granite emplacement

This section describes a two-dimensional forward model of intrusion emplacement, to demonstrate that the temperature in the country rocks near the intrusion margin accurately captures the temperature within the granite body. This modelling is conduced on a 2D slice, and Figure 4 schematically shows the geometrical relationship between the intrusion, this slice, and the 1-dimensional profile of our observations and inversions in the paper. We model the top 15 km of the crust; the surface is kept at 0 °C, and the base has a constant heat flux that results in a pre-intrusion steady-state thermal gradient of 30 °C/km (Figure 5a). The left- and right-hand boundaries are reflection boundary conditions (i.e. dT/dx = 0). The right-hand boundary is distant enough from the intrusion (at 25 km) that the choice of boundary condition does not affect the results.

We use a purely kinematic model, in which an intrusion is emplaced into the lower left corner of the model (dashed line on Figure 4) by upwards motion from the bottom boundary at a given rate, which we vary between model runs (black arrow on Figure 5a). We vary the functional form of the input velocity profile, to simulate: (1) the intrusion of an rigid granite, accompanied by shear in a thin band on its margin (Figure 5); (2) intrusion of a rigid granite, accompanied by spatially-distributed shear on its margin (Figure 6); (3) intrusion of a deforming granite, accompanied by spatially-distributed shear on its margin (Figure 7). The velocity distribution as a function of horizontal distance is shown on panel c of each Figure. For case (2), given the range of deformation patterns in the wall rocks adjacent to plutons that can be formed depending on rheology (e.g. Newtonian vs. non-Newtonian flow, see ref 33 in main paper) and the temperature-dependence of viscosity, we use a simple functional form in which the velocity decays as $v = A/x$ with distance from the intrusion. The constant A is chosen to result in most shear occurring in a band of a similar width to that of the granite (Figure 6c). For case (3), we use a simple Sine curve to parameterise the velocity distribution through the granite and country rocks (Figure 7c) – although in reality the precise functional form of this curve would depend upon the same rheological parameters as discussed above, for both the granite and country rocks. The same velocity profile is used throughout the thickness of the model domain, equivalent to the upwards motion of the column containing the granite being balanced by erosion at the surface. Whilst not realistic in detail, the precise distribution of velocity in the region above the granite has no significant influence on our results.

On the lower model boundary where the intrusion enters the model domain, the temperature is fixed at 800 °C. The intrusion can then change temperature by advection and diffusion once within the model domian. The models are run for a length of time corresponding to the time taken for emplacement (set by the velocity of intrusion), plus five times the characteristic diffusive thermal time constant for a 6 km wide intrusion (calculated as $\tau = l^2/\pi^2\kappa$, where l is the intrusion width and κ is the thermal diffusivity). This

duration ensures that all points within our area of interest have achieved their maximum temperature, and are cooling by the end of the model run. The intrusion width is 3 km, and given that this represents half of the intrusion adjacent to the reflection boundary condition, the full intrusion width is 6 km. The intrusion thickness is chosen to match this value (i.e. 6 km), and the depth from the surface to the top of the final intrusion emplacement level is 9 km. All of these parameters were chosen to approximate the values at Skiddaw. The temperature in the model evolves by diffusion, and by advection when the intrusion is being emplaced.

Figure 5a shows an example model run, displaying the maximum temperature experienced at each point and at any time. The difference between the temperature of the intrusion at any given crustal level and the temperature in the adjacent aureole can be visualised by examining temperature contrasts along horizontal planes through the model (Figure 4). Figure 5b shows an analysis of the results, expressed as the value of the maximum temperature experienced in the innermost part of the aureole as a proportion of the total temperature difference between the outer portion of the intrusion at the same crustal level and the pre-intrusion country rock. Instantaneous intrusion would result in a value of 0.5, i.e. the temperature would be mid-way between the intrusion and background values. Because of the heat exchange between the intrusion and country rocks during transport and emplacement, the innermost part of the aureole reliably records the temperature in the intrusion (to within a few percent) for the geologically-reasonable intrusion rates we have tested (1–100 mm/yr). Figures 6 and 7 show the equivalent patterns for other imposed velocity distributions. The precise details of the distribution of maximum temperatures attained depends upon the imposed velocity distribution, but the similarity between the model results presented here shows that temperature transport away from the granite during intrusion has a larger effect than the degree to which wall rocks are advected vertically along with the granite.

This result is as expected, because the thermal timescale for heat exchange between the granite and the aureole (months, estimated as $\tau = l^2/\pi^2\kappa$, where l is on the order of tens of metres for our closest samples) is short compared to the timescale required for intrusion (i.e. more then ten thousand years, to travel over 1 km at rates of 100 mm/yr or lower). Therefore, a given point within the aureole will experience protracted heat input from the granite as it is transported through that crustal level. Likewise, the granite will cool during intrusion by sustained contact with the aureole. The end result is that the proximal aureole samples reliably record the temperature of the adjacent granite. Although the each parcel of granite will have been hotter at some time, when at depth, what we are aiming to test is whether the country rocks accurately record the temperature at which granite was emplaced adjacent to them, at their crustal level. These numerical tests show this to be the case, meaning that we can use our metamorphic results to investigate the temperature, and so mechanism, of emplacement.

5 Evolution of temperature with space and time

Figure 8 shows the evolution of temperature with space and time, for the two models shown in Figure 2a in the main paper.

6 Pluton with dipping margin

Figure 9 shows results equivalent to those in Figure 2 in the main paper, but with the distances between the intrusion margin and the sample locations re-calculated assuming that the intrusion margin dips at $60°$.

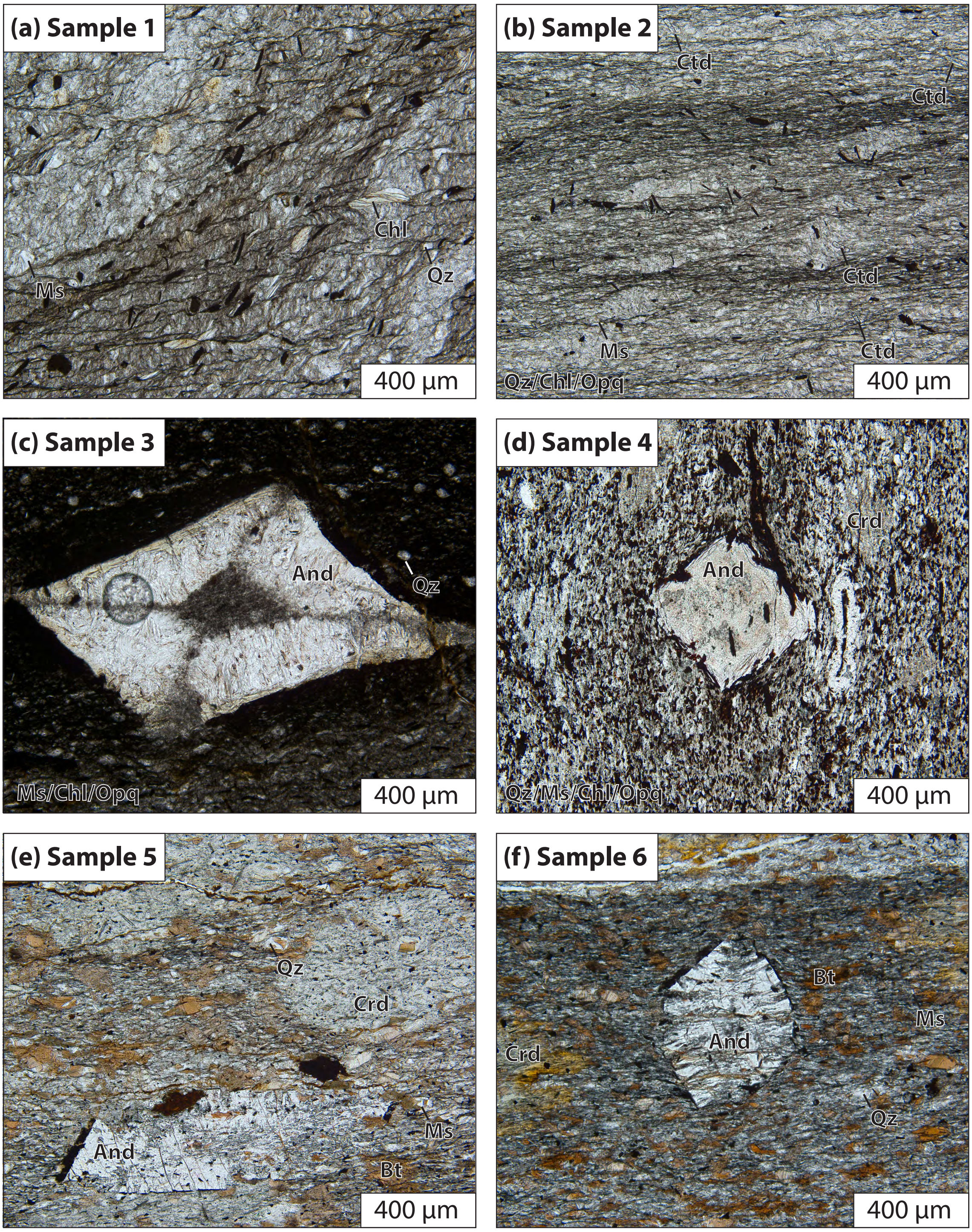

Figure 1: Petrography 1. (a–f) Plane-polarised light photomicrographs of samples 1–6. All images are at the same scale for ease of comparison. Only major phases are labelled. See supplementary text for discussion.

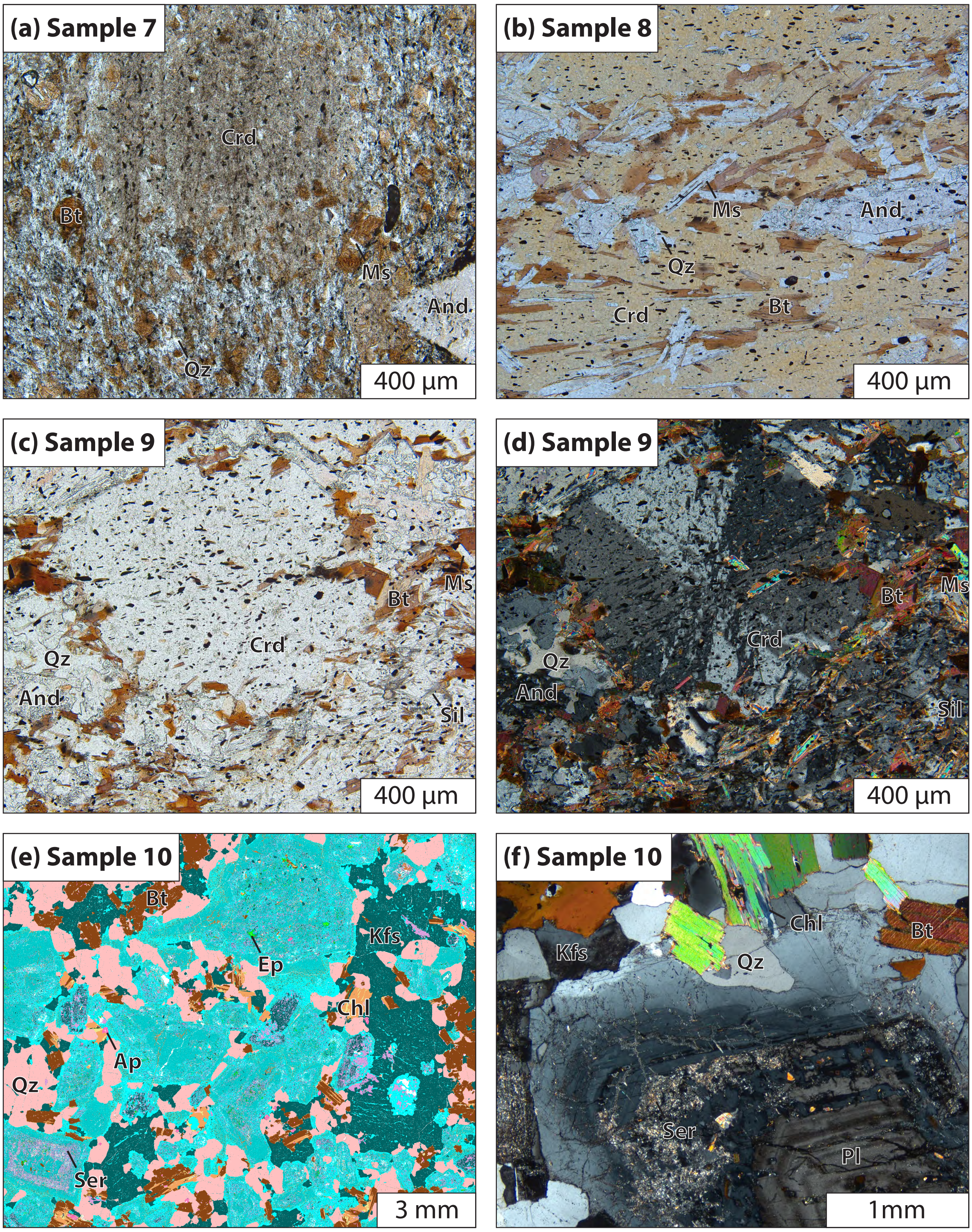

Figure 2: Petrography 2. (a–c) Plane-polarised light photomicrographs of samples 7–9. All images are at the same scale for ease of comparison. (d) As (c), but viewed under crossed polars to highlight the sector trilling in cordierite. (e) QEMSCAN image of sample 10 showing porphyritic texture. (f) Photomicrograph viewed under crossed polars of an oscillatory and concentrically zoned plagioclase grain in sample 10. See supplementary text for discussion.

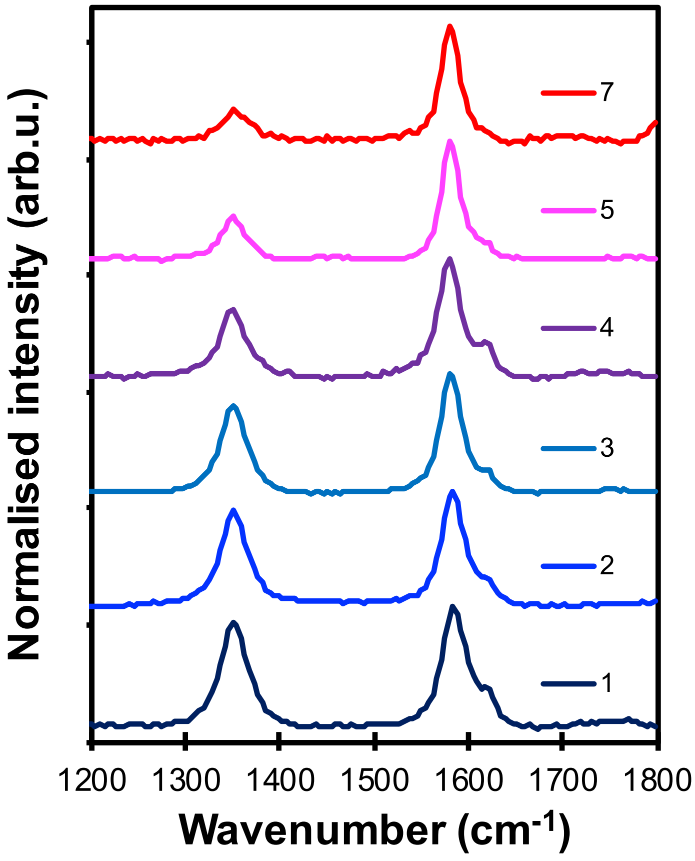

Figure 3: Average Raman spectra for graphite in samples 1, 2, 3, 4, 5 and 7.

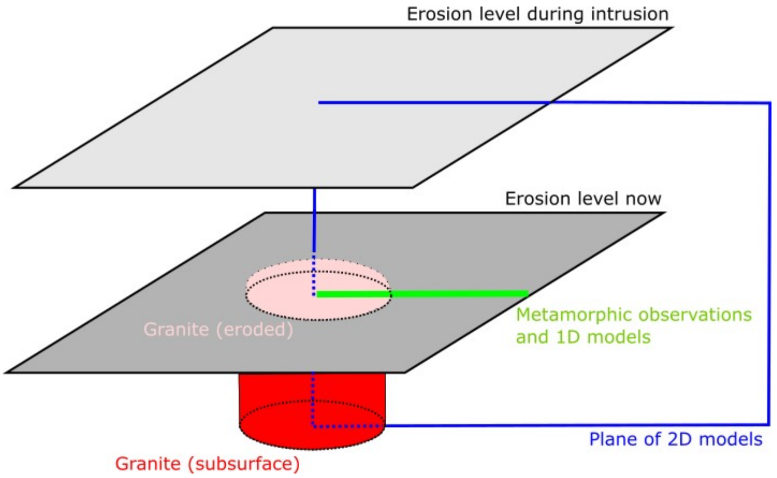

Figure 4: Schematic representation (not to scale) of the geometrical relationship between the intrusion, the slice used for the 2-dimensional models in this supplement, and the 1-dimensional profile of our observations and inversions in the paper. The outline of the 2D plane is dashed where within the granite.

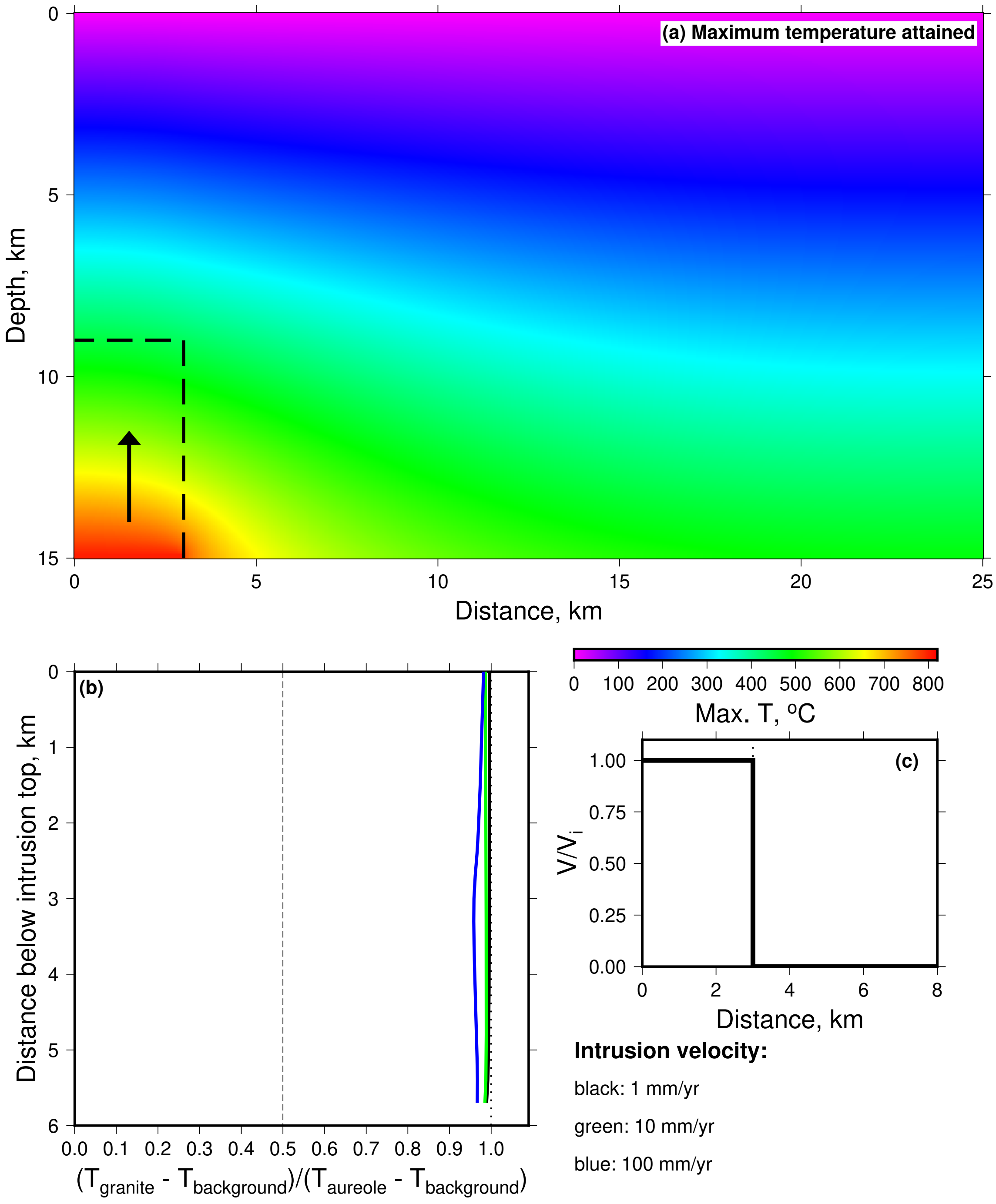

Figure 5: (a) Forward model of intrusion, as described in the text. The dashed black outline shows the final geometry of the granite (the left-hand edge of the model is a reflection boundary condition). The colours represent the maximum temperature experienced at each point, at any time during the model run. This model is for a 1 mm/yr intrusion velocity. (b) shows the value of the maximum temperature experienced in the innermost part of the aureole as a proportion of the total temperature difference between the outer portion of the intrusion temperature at the same crustal level and the pre-intrusion country rock temperature. Panel (c) shows the horizontal variation of vertical velocity during intrusion, normalised to the imposed maximum intrusion velocity (V_i), which is varied between 1 mm/yr, 10 mm/yr, and 100 mm/yr, and shown as different coloured lines on panel (b).

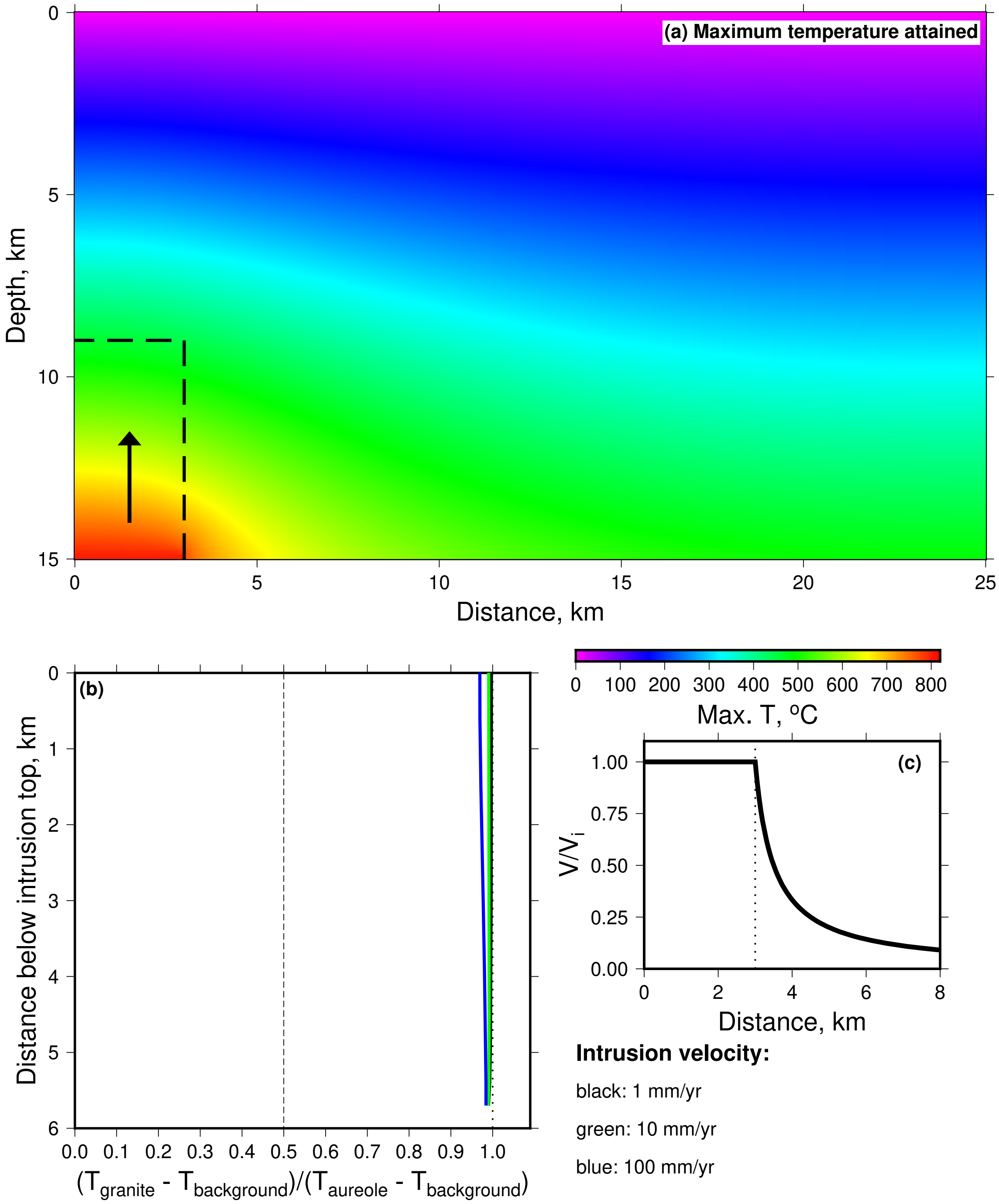

Figure 6: As Figure 5, but with a different profile of velocity during intrusion (see panel (c) and text).

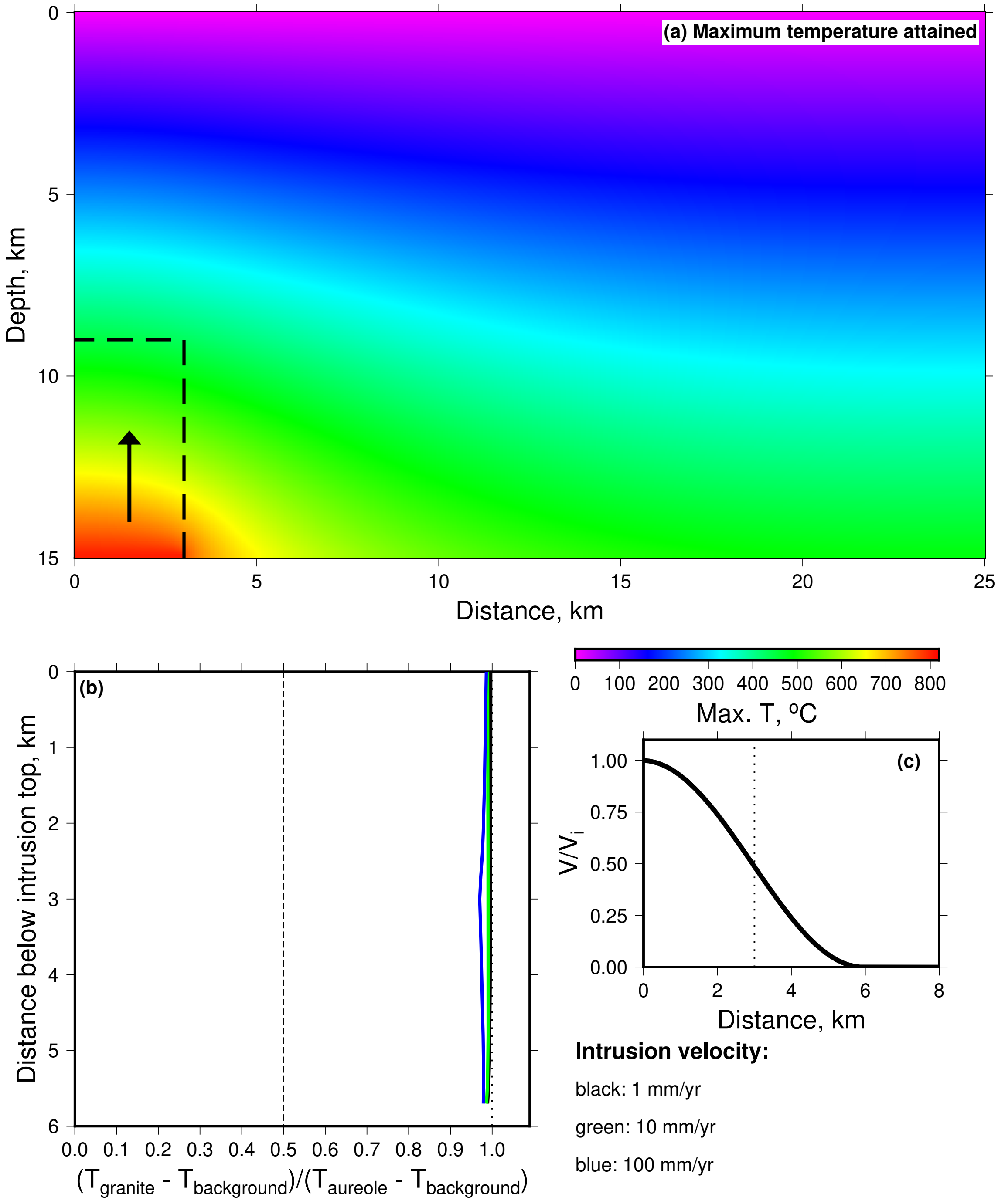

Figure 7: As Figure 5, but with a different profile of velocity during intrusion (see panel (c) and text).

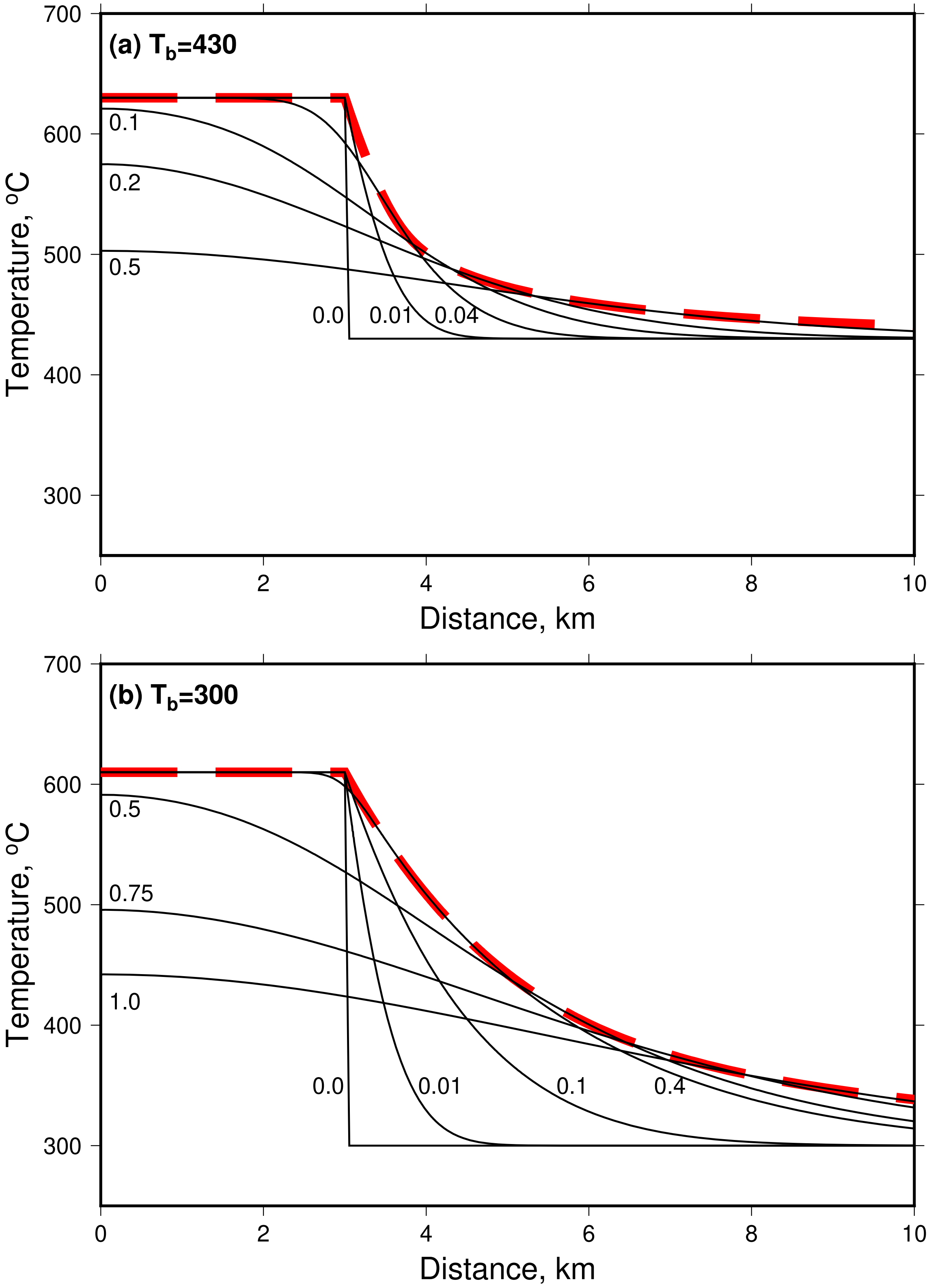

Figure 8: The evolution of temperature with space and time, for the models whose maximum temperatures are shown in Figure 2a in the main paper. The red lines show the curves plotted on Figure 2a, and the black lines show snapshots of the temperature field, labelled with the time after intrusion begins in Myr.

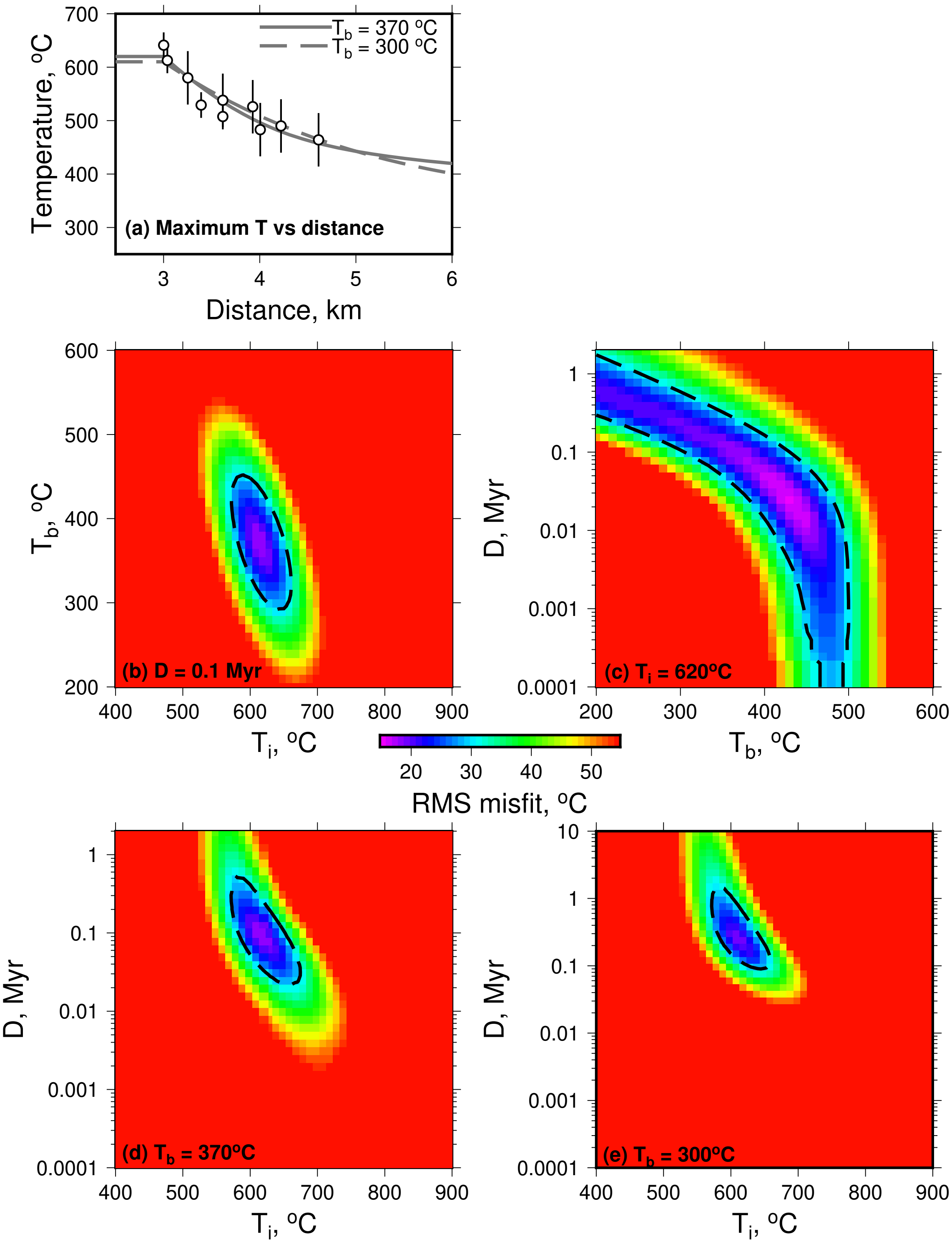

Figure 9: As Figure 2 in the main paper, but with distances re-calculated assuming that the pluton margin dips at 60°.
